# Lead Halide Perovskites Nanocrystals Synthesized in a Green, Reusable Solvent

**DOI:** 10.1002/smll.202500535

**Published:** 2025-05-02

**Authors:** Davide Pratolongo, Marta Campolucci, Marco Vocciante, Lorenzo Pugliesi, Emmanuela Di Giorgio, Chiara Lambruschini, Liberato Manna, Federico Locardi

**Affiliations:** ^1^ Dipartimento di Chimica e Chimica Industriale Università degli Studi di Genova Via Dodecaneso 31 Genova 16146 Italy; ^2^ Electron Microscopy Facility Istituto Italiano di Tecnologia Via Morego 30 Genova 16163 Italy; ^3^ Nanochemistry Department Istituto Italiano di Tecnologia Via Morego 30 Genova 16163 Italy

**Keywords:** colloidal nanocrystals, green solvent, lead halide perovskites, life cycle assessment, limonene

## Abstract

The widespread development of nanomaterials calls for searching for alternative processes that can reduce the impact, both from an environmental and economic point of view, of the syntheses employed to prepare such materials. Here, we report the synthesis of CsPbX_3_ (X = Cl, Br, I) nanocrystals using (*R*)‐ and (*S*)‐limonene, two molecules extracted from natural sources, as possible solvents. The nanocrystals prepared in this solvent have structural, optical, and morphological properties that are comparable to those of their homologs synthesized using more traditional solvents, i.e., 1‐octadecene. The relatively high volatility of limonene is then exploited for its recovery from the waste of the reactions and its reuse for subsequent syntheses. The replacement of 1‐octadecene with limonene as the reaction solvent is carefully evaluated by examining the consequences of the environmental impact of the entire synthesis process through a life cycle assessment procedure (CO_2_ footprint analysis), aiming at verifying any gain in Global Warming Potential reduction and identifying the most environmentally impactful steps of the process.

## Graphical abstract

Limonene has been employed as a green and reusable solvent for the synthesis of colloidal nanocrystals. This solvent can be recovered after the synthesis and reused. The environmental impact of the solvent has been estimated through a life cycle assessment.

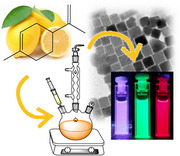

## 1 Introduction

Colloidal nanocrystals (NCs) have been known for 30 years. They have revolutionized the chemistry, physics, and materials science communities, with a strong impact on technology, as corroborated by the assignment of the 2023 Nobel Prize in Chemistry for the discovery and synthesis of Quantum Dots.^[^ ^1^ ^]^ Nowadays, NCs are employed in several technological fields such as displays, solar cells, lasers, and magnets, just to mention a few.^[^ ^2^, ^3^ ^]^ Their synthesis usually follows a hot‐injection approach, in which the nucleation of the NCs is triggered by the quick injection of a mixture of reagents in a hot mixture of additional reagents and of ligands dispersed in an organic solvent.^[^ ^4^, ^5^, ^6^, ^7^, ^8^, ^9^, ^10^, ^11^, ^12^, ^13^, ^14^, ^15^, ^16^ ^]^ Tri‐n‐octylphosphine oxide (TOPO) is the traditional solvent (acting also as a ligand) employed for the synthesis of Cd chalcogenides NCs, whereas for example 1‐octadecene (ODE) and dioctylether (DOE) are used in the synthesis of halide perovskites NCs.^[^ ^17^ ^]^ Diphenyl ether (DPE) is often employed for the synthesis of magnetic NCs.^[^ ^18^, ^19^, ^20^ ^]^ In principle, a good solvent for the hot injection synthesis needs to be non‐polar or only moderately polar, stable at high temperatures, relatively inert under the synthesis conditions, and with a high boiling point. For example, ODE boils at 320 °C and has a dielectric constant (ε) of 2.246 at 25 °C.^[^ ^21^ ^]^ Despite the widespread use of solvents such as TOPO, ODE, DOE, DPE, and others, various studies have identified critical issues that may affect the final products. TOPO tends to decompose under prolonged heating, leading to impurities such as alkylphosphinic acid.^[^ ^22^, ^23^, ^24^, ^25^ ^]^ ODE can polymerize during the synthesis, ending up embedding the final NCs in a polymer matrix that is difficult to separate from the NCs.^[^ ^26^, ^27^ ^]^ DPE can decompose into volatile by‐products^[^ ^28^ ^]^ that can affect the NCs morphology by reacting with the surface of the growing NCs. Moreover, the high boiling points of all the mentioned solvents used in the syntheses requires repeated washing (precipitation of the nanocrystals and resuspension in another solvent) to get rid of them at the end of the syntheses in order to obtain sufficiently clean NC samples. Finally, all the solvents mentioned above derive from petroleum and/or the synthetic industry and are discharged after each synthesis, with the consequent production of large volumes of chemical waste.

The use of the so‐called green solvents is established in organic chemistry, where deep eutectic solvents (DESs)^[^ ^29^, ^30^ ^]^ and ionic liquids (ILs)^[^ ^31^, ^32^ ^]^ have been proposed as alternative solvents and are now largely used in reactions as modifications of the functional groups, organometallic catalysis, enzymes catalysis, multicomponent reactions,^[^ ^30^, ^33^, ^34^, ^35^, ^36^ ^]^ and in extraction procedures. DESs and ILs have also been used in the NCs synthesis: Lu et al. and Chatterjee et al. suggested a synthesis of lead halide perovskites (LHP) NCs via ligand‐assisted reprecipitation (LARP) using DESs based on menthol^[^ ^37^, ^38^ ^]^ and thymol.^[^ ^38^ ^]^ Similarly, Chen et al. employed the IL 1‐methyl‐3‐methylimidazolium bromide in the synthesis of CsPbBr_3_ NCs.^[^ ^39^ ^]^ Additional recent studies^[^ ^40^ ^]^ have explored the possibility of using ODE in combination with biogenic organic ligands, e.g. creatine phosphate,^[^ ^41^ ^]^ or with ligands derived from natural sources, e.g. soy lecithin.^[^ ^10^ ^]^

**Figure 1:**
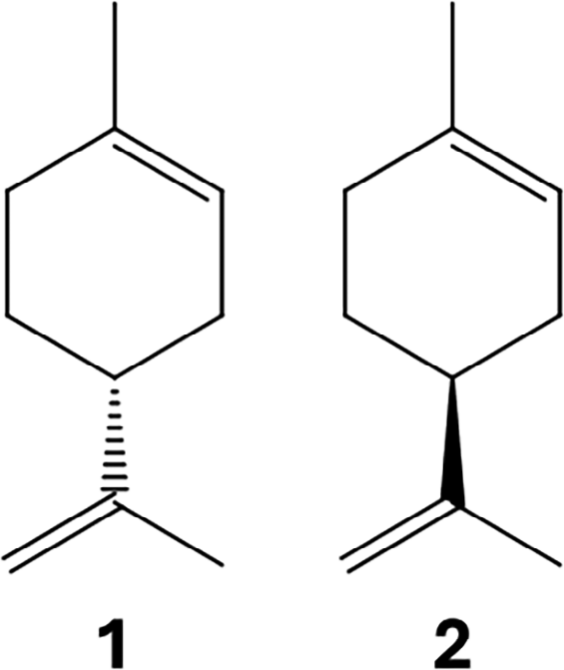
Molecular structure of (**1**) (R)‐ and (**2**) (S)‐limonene.

In this work, we have explored the synthesis of LHP NCs using limonene (1‐methyl‐4‐(prop‐1‐en‐2‐yl)cyclohex‐1‐ene) instead of more conventional solvents. Limonene, the principal component of the essential oil of the citrus peels, is a monoterpene that exists in two different enantiomers, (*R*)‐ (**1**) and (*S*)‐ (**2**), respectively (**Figure** **1**). Generally, the essential oil from citrus species contains a predominance of (*R*)‐limonene whereas the enantiomer (*S*)‐ is found in spearmint, peppermint, citronella, and lemongrass.^[^ ^42^, ^43^ ^]^ However, (*S*) ‐limonene has a relatively low abundance that reaches a maximum of 5%, so it is generally synthesized from α‐pinene, a molecule extracted from pines.^[^ ^44^ ^]^ (*R*)‐limonene on the other hand is easily obtained in high enantiomeric excess by extraction from orange or lemon peels, which are abundant by‐products of the agri‐food industry. Today, limonene is considered a green solvent and has been suggested as a greener alternative in several processes.^[^ ^45^, ^46^, ^47^ ^]^

By employing either (*R*)‐ or (*S*)‐limonene as a solvent, we successfully synthesized CsPbCl_3_, CsPbBr_3,_ and CsPbI_3_ NCs with control over size, morphology, and optical properties. Their overall features (size homogeneity, crystallinity, optical absorption, and emission spectra) were generally comparable to those of the corresponding NCs prepared with ODE, while photoluminescence quantum yield (PLQY) values could be better (for CsPbBr_3_), comparable (CsPbI_3_) or worse (CsPbCl_3_). In the CsPbCl_3_ case, one major cause for the lower PLQY was the synthesis temperature required by the lower boiling point of limonene compared with ODE. a remarkably valuable aspect of limonene is that we could even vacuum‐distill it from the final product as a relatively pure solvent and reuse it for a new synthesis, thus demonstrating its recyclability. Finally, following a life cycle assessment (LCA) based on CO_2_ footprint analysis, we identified the actual gain in terms of Global Warming Potential (GWP) and highlighted the lower impact of the CsPbBr_3_ and CsPbI_3_ synthesis using limonene instead of ODE.

## 2 Results and Discussion

The CsPbX_3_ (X = Cl, Br, I) NCs were synthesized according to established syntheses, with ODE being replaced with either (*R*)‐ or (*S*)‐limonene (see Supporting Information and Tables S1–S4, Supporting Information). Limonene was selected as a solvent considering its relatively lower boiling point (≈175 °C) compared to that of ODE (≈300 °C). Although this choice entails a narrower synthesis temperature range, the lower boiling point of limonene can be exploited both to recycle and quantitatively remove it from the NCs at the end of the synthesis (vide infra).

### 2.1 CsPbCl_3_ NCs

**Figure 2:**
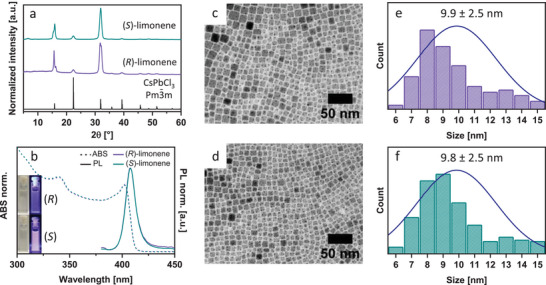
a) XRD patterns, and b) optical absorption (dash line) and PL (solid line, λ_exc_ = 365 nm) spectra of CsPbCl_3_ NCs synthesized in (R)‐limonene (purple color) and (S)‐limonene (dark cyan color); the insets in (b) are photographs of the colloidal dispersions under visible and 365 nm light irradiation; c,d) low‐magnification transmission electron microscopy (TEM) images and e. f) size distributions of the CsPbCl_3_ NCs synthesized in (R)‐limonene (c,e) and (S)‐limonene (d,f).

The CsPbCl_3_ NCs were synthesized according to Imran et al.,^[^ ^48^ ^]^ with the only exceptions being that degassing was carried out at room temperature and the injection temperature was decreased from 200 to 187 °C, as the latter is the temperature at which the reaction was at reflux (details of the synthesis are reported in the Experimental Section). The increase of this temperature with respect to the boiling point of the pure solvent is ascribable to the presence of the high boiling point ligand molecules. After the purification steps, the final colloidal dispersion was colorless and emitted a feeble violet light under UV irradiation (**Figure** **2**). According to X‐ray diffraction (XRD) analysis (Figure 2a), the NCs had a $\mathrm{Pm}\bar{3}m$ cubic structure, as expected.^[^ ^49^ ^]^

The differences in the relative intensities of the peaks compared to the bulk pattern are ascribed to a preferential orientation of the cubic NCs on the substrate, as extensively reported in the literature. Moreover, the first diffraction peak splits into two signals, evidencing the formation of an ordered superlattice on the as discussed by some of us in previous works.^[^ ^50^, ^51^, ^52^ ^]^ The optical absorption spectra (ABS) of the NCs (Figure 2b) were characterized by an excitonic peak at 400 nm, whereas the photoluminescence (PL) spectra evidenced a well‐defined signal at 408 nm. The full widths at half maximum (FWHM) of the PL peaks were 85 and 83 meV for NCs synthesized in (*R*)‐ and (*S*)‐limonene, respectively (Figure S1, Supporting Information). The synthesis in (*R*)‐limonene delivered cubic‐shaped NCs with an edge length of 9.9 ± 2.5 nm; similar results were obtained in (*S*)‐limonene where the NCs were also cubic in shape, with an edge length of 9.8 ± 2.5 nm (Figure 2c–f and **Table** **1**). The PLQY values were 2% and 5% for the samples prepared in (*R*)‐ and (*S*)‐limonene, respectively, which are lower than those of CsPbCl_3_ NCs prepared with more traditional methods. We ascertained that one reason is certainly the synthesis temperature. Indeed, switching back to ODE as a solvent and lowering the temperature of the synthesis from 200 to 187 °C caused a decrease in the PLQY of the NCs from 34% to 20% (Figure S2 and Table S5, Supporting Information).

**Table 1:** Comparison of CsPbX_3_ NCs synthesized in (*R*)‐ and (*S*)‐limonene in terms of NCs size and optoelectronic properties.

Stoichiometry	Solvent	T_inj_	Size [nm]	PLQY	Peak wavelength	FWHM of PL
CsPbCl_3_	(*R*)‐limonene	187 °C	9.9 ± 2.5	2%	408 nm	85 meV
	(*S*)‐limonene	187 °C	9.8 ± 2.5	5%	408 nm	83 meV
CsPbBr_3_	(*R*)‐limonene	160 °C	9.2 ± 1.0	86%	513 nm	75 meV
	(*S*)‐limonene	160 °C	9.1 ± 1.1	86%	513 nm	74 meV
CsPbI_3_	(*R*)‐limonene	165 °C	17.0 ± 2.0	53%	692 nm	81 meV
	(*S*)‐limonene	165 °C	17.2 ± 1.8	66%	692 nm	81 meV

### 2.2 CsPbBr_3_ NCs

**Figure 3:**
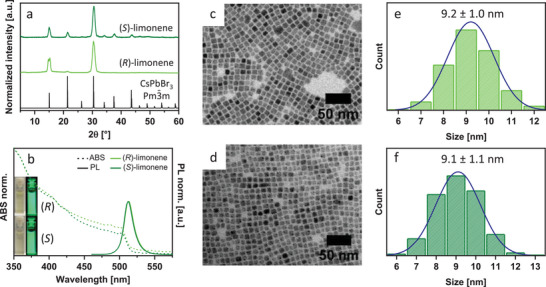
a) XRD patterns, and b) optical absorption (dash line) and PL (solid line, λ_exc_ = 365 nm) spectra of CsPbBr_3_ NCs synthesized in (*R*)‐limonene (green color) and (*S*)‐limonene (olive‐green color); the insets in (b) are photographs of the colloidal dispersion under visible and 365 nm light irradiation; c,d) low‐magnification TEM micrograps and e,f) size distribution of the CsPbBr_3_ NCs synthesized in (*R*)‐limonene (c,e) and (*S*)‐limonene (d,f).

The CsPbBr_3_ NCs were synthesized following the method proposed by Baranov et al.^[^ ^53^ ^]^ in our case working under nitrogen instead of air (details of the synthesis are reported in the Experimental Section). Briefly, the synthesis consisted of a hot‐injection method in which PbBr_2_, oleyl amine (OLAM), oleic acid (OLAC), and limonene were mixed in a vial under an inert atmosphere. Then, the temperature was increased and at 160 °C the cesium oleate (CsOLAC) solution was swiftly injected, followed by natural cooling. Additional details are reported in the Experimental Section. The XRD analyses of the final, purified sample confirmed the orthorhombic $\mathrm{Pm}\bar{3}m$ structure for the NCs prepared both in (*R*)‐ and (*S*)‐ limonene (**Figure** **3**a).^[^ ^54^ ^]^ As for the CsPbCl_3_ discussed earlier, the two most intense peaks, related to the (1,0,0) and (2,0,0) crystallographic planes, reflect the tendency of the NCs to orient preferentially on the sample holder. Figure 3b reports the ABS and PL spectra. In the ABS the excitonic peak was at 502 nm for both samples; the emission was dominated by a sharp peak centered at 513 nm with a FWHM of 75 meV and 74 meV for the NCs prepared in (*R*)‐ and (*S*)‐limonene, respectively (Figure S1, Supporting Information). Interestingly, both materials had a remarkably high PLQY (≈85%), which is higher than that of the same NCs synthesized in ODE (Table S5, Supporting Information). The TEM micrographs confirmed the cubic shape of the NCs (Figure 3c,d) and a good size distribution for both the samples (Figure 3e,f), with 9.2 ± 1.0 and 9.1 ± 1.1 nm for (*R*)‐ and (*S*)‐limonene, respectively. The mean size is consistent with the peak position in the PL spectra.^[^ ^55^ ^]^ Similar results were obtained for the NCs prepared in ODE (Table S5 and Figure S3, Supporting Information) except for their lower PLQY, although a modification of the injection temperatures leads to NCs with broader size distribution (Figure S4, Supporting Information).

### 2.3 CsPbI_3_ NCs

**Figure 4:**
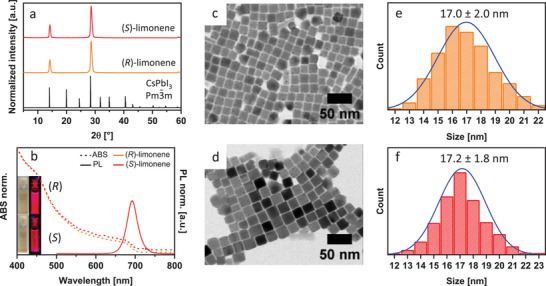
a) XRD patterns, and b) optical absorption (dash line) and PL (solid line, λ_exc_ = 365 nm) spectra of CsPbI_3_ NCs synthesized in (R)‐limonene (orange color) and (S)‐limonene (red color); the insets in (b) are photographs of the colloidal dispersion under visible and 365 nm light irradiation; c,d) low‐magnification TEM micrographs and e,f) size distribution of the CsPbI_3_ NCs synthesized in (R)‐limonene (c,e) and (S)‐limonene (d,f).

The CsPbI_3_ NCs were synthesized by the method proposed by Akkerman et al.^[^ ^56^ ^]^ but working under nitrogen instead of air (details of the synthesis are reported in the Experimental Section). The synthesis consists of a hot‐injection method in which an oleylammonium iodide (OLAM‐I) precursor is injected, triggering the NCs formation (see the Experimental Section). After the purification steps, the final colloidal dispersions have a red color and emit red light under UV excitation (**Figure** **4**).

The XRD analyses indicated a structure compatible with that of bulk cubic CsPbI_3_ (Figure 4a),^[^ ^57^ ^]^ however with only some peaks present, due to preferential orientation. The features of these NCs are very similar to those of the same NCs synthesized in ODE (Figure S5, Supporting Information). Similarly to the Cl and Br‐based NCs, the absorption and emission spectra of the NCs did not depend on which limonene enantiomer was used (Figure 4b). The absorption was characterized by an excitonic peak at 665 nm whereas the emission was peaked at 692 nm; the FWHM of the emission peak was 81 meV and 81 meV for the NCs synthesized in (*R*)‐ and (*S*)‐limonene, respectively. Figure 4c,d are TEM micrographs of the samples. They reveal a cubic shape of the NCs, with an edge length of 17.0 ± 2.0 nm for (*R*)‐limonene and 17.2 ± 1.8 nm for (*S*)‐limonene (Figure 4e,f). The PLQY was 53% ((*R*)‐limonene) and 66% ((*S*)‐limonene). These values are comparable with those of the same NCs prepared in ODE (Table S5, Supporting Information), even if the sample synthesized in ODE at the same temperature as the synthesis in limonene (165 °C) was characterized by a double population formed by cubes and rods (Figure S5, Supporting Information); the same behavior was observed by reducing the injection temperature to 150 °C when limonene was used (Figure S6, Supporting Information).

To test the stability of the synthesized NCs, the pristine materials were stored for 31 days at 4 °C and then analyzed. All the NCs retained their structural, morphological, and optical properties (Figures S7–S9, Supporting Information); however, we observed a decrease in the PLQY both in the sample synthesized in ODE and in limonene (Table S6, Supporting Information).

### 2.4 Solvent Recovery and Stripping

As already anticipated, limonene is characterized by a relatively low boiling point (≈175 °C); even if this limits the temperature range achievable during the synthesis, on the other hand it allows solvent recycling and, in principle, its complete removal from the NCs. The waste obtained after the synthesis is a colored mixture (Figure S10a, Supporting Information) constituted by the solvent, the ligands (OLAM and OLAC), and possible unreacted inorganic salts. OLAM and OLAC have a high boiling point and a low vapor pressure; the metals and their derivates are practically non‐volatile. We then performed a vacuum distillation (Figure S10b, Supporting Information) to check the possibility of retrieving pure limonene (see Experimental Section). The recovered liquid (Figure S10c, Supporting Information) was clear and colorless for the (*R*)‐limonene case and clear and pale yellow for the (*S*)‐limonene case. The distillation yield was quantified in 70% and 90% for the (*R*)‐ and (*S*)‐ enantiomers, respectively. The ^1^H NMR spectra of the two liquids confirmed the purity of the distillates and the absence of secondary organic products (Figure S10d, Supporting Information). The possible presence of Cs and Pb was excluded by the ICP–AES and ICP–MS analyses (the concentration of both elements was lower than the detection limit, see the Supporting Information). We then employed the recovered solvents to synthesize the CsPbX_3_ NCs again. The NCs prepared from this second synthesis using the recycled solvents had the same features as the NCs obtained in the original pure solvents (Figures S11–S13, Supporting Information).

In the traditional syntheses using ODE, several studies have reported the issue of incomplete removal of ODE from the NCs even after several washing steps.^[^ ^26^, ^27^, ^58^ ^]^ We verified that limonene instead can be quantitatively removed from the NCs just by vacuum pumping. Indeed, we put a portion of CsPbBr_3_ NCs suspension in hexane under vacuum overnight; then, the NCs were dispersed in tol‐d_8_ and analyzed by ^1^H NMR. The analyses confirmed the complete removal of the limonene, differently from what was observed for the NCs prepared in ODE (Figure S14, Supporting Information).

### 2.5 LCA Comparison Between the Different Synthetic Paths

The aforementioned results highlight how limonene can be successfully used for the synthesis of LHPs NCs, especially for the Br and I‐ based compounds. To investigate the green aspect of our approach, we performed a dedicated analysis using an LCA approach. **Table** **2** shows the environmental impacts (GWP) in terms of kg of CO_2_ equivalent released during the synthesis of 100 g of NCs. These values were calculated using the value reported in Table S7 (Supporting Information) and the reaction yields (Table S8, Supporting Information).

**Table 2:** GWP values for the synthesis of 100 g of NCs. The values in brackets refer to the distilled limonene.

Phase	Material/Operation	GWP [kg_CO2eq_/100 g CsPbCl_3_]		GWP [kg_CO2eq_/100 g CsPbBr_3_]		GWP [kg_CO2eq_/100 g CsPbI_3_]	
		ODE	LIM	ODE	LIM	ODE	LIM
reaction	Solvent	4.52	1.91 (0.57)	42.4	7.12 (2.14)	9.39	2.83 (0.85)
	Pb source	0.07	0.18	*	**	0.15	0.28
	Cs source	0.03	0.08	3.84	4.05	0.06	0.12
	Halogen source	0.34	0.89	1.17*	1.23**	1.11	2.10
	Oleic compounds	0.35	0.93	1.27	1.34	0.12	0.23
sub total		5.31	3.99 (2.65)	48.7	13.8 (8.8)	10.83	5.56 (3.58)
aux	Distillation	–	– (1.34)	–	– (4.98)	–	– (1.98)
	Disposal	5.46	14.48 (9.10)	98.19	103.5 (83.5)	14.69	27.83 (19.84)
total		10.77	18.47 (13.09)	146.89	117.7 (97.28)	25.52	33.39 (25.4)

**Figure 5:**
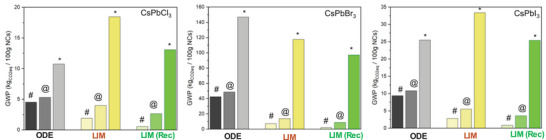
GWP values for the synthesis of 100 g of CsPbX_3_ (X = Cl, Br, I) estimated considering (#) only the solvent contribution, (@) the solvent and reagents, (^*^) and the solvent, reagents, distillation, and disposal.

The replacement of ODE with limonene leads to a reduction in CO_2_ emissions that ranges from 58% (CsPbCl_3_ in Table 2) to 83% (CsPbBr_3_ in Table 2); for CsPbBr_3_, the reduction reaches up to 95% for the synthesis of CsPbBr_3_ NCs if limonene is recovered by distillation (42.4 GWP and 2.14 GWP for the ODE and limonene, respectively; Table 2 and **Figure** **5**). If we consider limonene as a raw material, the benefit obtainable by its recovery it is offset by a substantially equivalent contribution related to the impact of the distillation process. Since limonene is produced industrially by distillation, the impact is fully consistent with the production from scratch. However, the benefit of this process becomes clear when considering the drastic reduction in the volume of the disposal solution.

Comparing the impact of the three syntheses (reaction phase), the use of limonene as a solvent leads to a measurable reduction in the carbon footprint (by ≈25%, 70%, and 50% for CsPbCl_3_, CsPbBr_3_ and CsPbI_3_, respectively; Figure 5). However, based on the reaction yield of CsPbI_3_, limonene is a less efficient solvent if one takes into account the greater use of chemicals required, all other conditions being equal. On the other hand, the NCs prepared in limonene possess better properties in terms of size control when compared to their homologs prepared in ODE under the same experimental conditions (Figure 4; Figure S5, Supporting Information). The lower efficiency for limonene is even more marked in the synthesis of CsPbCl_3_ (Table 2). Overall, considering all the parameters (consumption of the chemical, waste disposal, distillation, etc.) that affect the total impact of the synthesis, limonene is preferred over ODE only for CsPbBr_3_. For CsPbI_3_ the two solvents have a comparable impact, whereas for CsPbCl_3_ ODE is actually preferred (Figure 5). The analysis highlighted significant differences in terms of overall impact among the syntheses, regardless of the solvent used, with the CsPbBr_3_ synthesis being by far the most impactful one. Finally, we also considered the influence of the washing step. Interestingly, the use of ethyl acetate (AcOEt the overall sustainability (Table S9, Supporting Information). Thus, for future process optimization, the replacement of AcOEt appears to be of primary importance.

## 3 Conclusion

In conclusion, we have successfully synthesized CsPbX_3_ (X = Cl, Br, I) NCs using the alternative and green solvents (*R*)‐ and (*S*)‐limonene. The obtained NCs presented a cubic morphology and had crystal structure and optical properties comparable to those of their homologs synthesized using the more traditional solvent ODE. By exploiting the relatively low boiling point of limonene, we demonstrated its easy removal from the synthesized NCs and the possibility of its recovery from the synthesis waste as a pure solvent that could be reused for subsequent syntheses. Through an LCA approach, we confirmed the benefit of the use of limonene. In principle, limonene can be extended to the synthesis of other metal halide NCs and even to NCs of other compositions. In general, green solvents represent a valid option to reduce the production of waste, thus leading to a reduced economic and environmental impact in the synthesis of nanomaterials.

## 4 Experimental Section

### Materials

Benzoyl chloride (BzCl, Acros Organic, 99%), cesium carbonate (Cs_2_CO_3_, Alfa Aesar, 99.9%), ethyl acetate (AcOEt, Sigma–Aldrich, >99.5%), hexane (Sigma–Aldrich, 99%), iodine (I_2_, Fluka, >99.8%), lead(II) acetate trihydrate (Pb(OAc)_2_×3H_2_O, ThermoFisher Scientific, 99.995%), lead(II) bromide (PbBr_2_, Sigma–Aldrich, 99%), (*R*)‐limonene (ThermoFisher Scientific, 96%), (*S*)‐limonene (Sigma–Aldrich, 96%), oleic acid (OLAC, Alfa Aesar, tech. 90%), oleyl amine (OLAM, ThermoFisher Scientific, 80%–90%), oleyl amine (OLAM, Sigma–Aldrich, 70%), toluene (Alfa Aesar, 99%), deuterated toluene (tol‐*d*_8_, Sigma–Aldrich, 99.6 atom % D). All the chemicals were employed without any further purification.

### CsOLAC Synthesis

Cs_2_CO_3_ (400 mg), and oleic acid (OLAC, 1.75 mL) were loaded in a 40 mL vial with either (*R*)‐ or (*S*)‐limonene (15 mL) and put under nitrogen flux.^[^ ^55^ ^]^ The mixture was heated at 100 °C under vigorous stirring until the solution became fully transparent. The final transparent yellow solution (0.15 m) was used for the synthesis of CsPbBr_3_ NCs.

### Synthesis of OLAM‐I

I_2_ (0.75 mg), OLAM (80%–90%, 4.5 mL), and either (*R*)‐ or (*S*)‐limonene (10.5 mL) were loaded into a 25 mL three‐neck flask and put under nitrogen.^[^ ^56^ ^]^ Then, the mixture was heated at 100 °C, leading to a red solution. The so obtained OLAM‐I solution (0.4 m I_2_) was transferred, when still hot, into a vial, without any further purification and was then stored under nitrogen.

### Synthesis of CsPbCl_3_ NCs

Pb(OAc)_2_×3H_2_O (76.0 mg), Cs_2_CO_3_ (16.0 mg), OLAC (0.3 mL), OLAM (70%, 1 mL) and either (*R*)‐ or (*S*)‐limonene (5 mL) were loaded into a 25 mL three‐neck flask.^[^ ^48^ ^]^ The solution was put under vigorous stirring and left under vacuum for 1 h. Then, the atmosphere was switched to nitrogen, and the reaction temperature was raised until reflux (187 °C) and BzCl (210 µL) were swiftly injected, triggering the nucleation and growth of the NCs. After 20 s, the flask was cooled in an ice bath. The final suspension was orange and cloudy (Table S1, Supporting Information), and was centrifugated at 4000 rpm for 10 min. The precipitate was centrifuged again for 1 min to remove additional solvent, gently washed twice using toluene (300 µL), and then dispersed in hexane (2 mL). The final colloidal suspension was clear and colorless.

### Synthesis of CsPbBr_3_ NCs

PbBr_2_ (72.4 mg), OLAM (80%–90%, 410.2 mg (500 µL)), OLAC (44.5 mg (50 µL)) and either (*R*)‐ or (*S*)‐limonene (5 mL) were loaded into a 20 mL vial.^[^ ^55^ ^]^ The reaction mixture was put under nitrogen under vigorous stirring and heated at 165 °C to dissolve all of the precursors. Then, at 160 °C, CsOLAC solution (0.15 m, 500 µL) was swiftly injected, and the flask was cooled naturally to room temperature (Table S2, Supporting Information). The reaction mixture was centrifugated at 4000 rpm for 5 min, and the precipitate was discharged. A solution of AcOEt with 5% in volume of the ligands (in a 10:1 OLAM:OLAC ratio) was dropped into the supernatant until it turns cloudy. Then, the resulting mixture was centrifuged at 4000 rpm for 5 min, the precipitate was collected and dispersed in hexane (1.5 mL). The washing process was performed twice, leading to a green, transparent colloidal suspension.

### Synthesis of CsPbI_3_ NCs

Pb(OAc)_2_×3H_2_O (75.9 mg), Cs_2_CO_3_ (16.3 mg), OLAC (183.4 mg (200 µL)) and the proper solvent ((*R*)‐ or (*S*)‐limonene, 5 mL) were loaded into a 20 mL vial and pumped to vacuum for 1 h.^[^ ^56^ ^]^ Then, the temperature was quickly raised up to 165 °C and the pre‐heated OLAM‐I solution (0.4 m I_2_, 2 mL) was swiftly injected. After 10s the reaction was quenched using a water bath, leading to a cloudy and red suspension (Table S3, Supporting Information). The latter mixture was centrifugated at 6000 rpm for 5 min and the precipitate was washed as described for the synthesis of CsPbCl_3_ NCs. The final NCs were then dispersed in 5 mL of hexane.

### Vacuum Distillation

The waste derived from the first centrifugation step of each synthesis was loaded into a flask with a Vigreux column on the top and pumped to vacuum (5 mmHg). The flask was heated at 140 °C, inducing the vaporization of the limonene (vapor temperature of 55 °C). The distilled limonene was clear and colorless with a yield that was at least 70% and 90% for (*R*)‐ and (*S*)‐limonene, respectively.

### Optical Absorbance Measurements

These were carried out on a Shimadzu UV–2600i spectrophotometer. The samples were diluted in hexane (3 mL) in a quartz cell (optical path 10 mm) to obtain an absorption of ≈0.3 at the bandgap. The spectra were acquired in the 300–700 nm range for the CsPbCl_3_ and CsPbBr_3_ NCs, and the 300–900 nm range for the CsPbI_3_ NCs.

### Photoluminescence (PL) and Photoluminescence Quantum Yield (PLQY) Measurements

The photoluminescence spectra were recorded using an Edinburgh FS5 Spectrofluorometer, equipped with a continuous Xe lamp as an excitation source. The PL spectra were measured at 𝜆_exc_ = 365, 357, and 350 nm for CsPbCl_3_, CsPbBr_3_, and CsPbI_3_, respectively (excitation slit width 3 nm, emission slit width 2 nm). The PLE spectra were measured at the PL maximum (excitation slit width 2 nm, emission slit width 3 nm). The PLQY was estimated using an integrating sphere (0.8 nm excitation slit width and 0.8 nm detection slit width). The analyses were carried out on diluted NC solutions dispersed in hexane in quartz cuvettes (path length of 1 cm).

### X‐Ray Diffraction (XRD) Analysis

The XRD patterns were collected by a Rigaku MiniFlex 600 diffractometer. The samples were prepared by drop casting the final colloidal solution obtained from the synthesis on a zero background Silica wafer. The diffractogram was acquired using the Cu K_α_ irradiation, in the range 5°–60°, step 0.02° and speed 2.5° min^−1^.

### Transmission Electron Microscopy (TEM)

TEM micrographs were acquired using a JEOL JEM‐1011 microscope equipped with a thermionic gun at 100 kV accelerating voltage. The samples were prepared by dropping dilute suspensions of NCs onto carbon‐coated 200 mesh copper grids. The image elaboration was performed using the software ImageJ.^[^ ^59^ ^]^

### Nuclear Magnetic Resonance (NMR) Spectrometry

NMR spectra were measured on a JEOL JNM‐ECZ400R at 400 MHz (1H) at 20 °C in CDCl_3_. The chemical shifts (δ) were expressed in parts per million relatives to tetramethylsilane (TMS) as internal standard (0.00 ppm). The data treatment was performed using the MestReNova 14 software.^[^ ^60^ ^]^

### Inductive Coupled Plasma (ICP‐OES and ICP‐MS) Elemental Analysis

Lead concentrations in the distilled solvents were estimated by ICP‐OES using an iCAP 7600 DUOICP‐OES spectrometer (ThermoFisher Scientific). 30 µL of each distilled solvent was mixed with 1 mL of aqua regia (HCl:HNO_3_ 3:1, final concentration 10% v/v). After 8 h, the solution was added to 10 mL of ultrapure water. Thus, the final solution was filtered by a 0.45 µm PTFE filter and analyzed. Cesium was analyzed by an ICP–MS using an iCAP‐TQ mass spectrometer (Thermofisher Scientific); the sample was prepared as ICP–OES, adding HNO_3_ at 1% v/v on the final solution. The limit of detection (LOD) was 0.3 ppb and 0.01 ppb for the Pb and Cs, respectively.

### Life Cycle Analysis (LCA)

The assessment of the environmental consequences of an activity could be achieved through an LCA, which generally included the following four different steps (ISO 14040 (2006) and ISO 14044 (2006)): i) identification of objectives and scope (definition of system boundaries, functional units, and required data); ii) Life Cycle Inventory (LCI): collection of data, with analysis of related balances (energy and mass) according to the objective and scope; iii) Life Cycle Impact Assessment (LCIA): conversion of the LCI into environmental impacts, including consumption of natural resources and possible health impacts; iv) Interpretation: detailed analysis of the results obtained and identification of possible improvements to mitigate environmental impacts. Although originally developed to assess the life cycle of a product, the LCA methodology was increasingly employed in assessing the impact of processes.^[^ ^61^ ^]^ In a similar vein, it can also be used to assess the sustainability of different synthesis pathways and thus identify the most environmentally acceptable option. All analyses were performed using the Ecoinvent 3.10 database;^[^ ^62^ ^]^ all results were expressed in terms of Global Warming Potential over a 100‐year period (GWP‐100), according to the CML2001–January 2016 method.^[^ ^63^ ^]^ GWP is a relative measure based on the quantification of greenhouse gas emissions; other parameters could have been used,^[^ ^64^ ^]^ but GWP was considered the most interesting in relation to the current situation of global warming and climate change.

To obtain an easily interpretable and sufficiently meaningful evaluation, 100 g of each final product was considered as a functional unit for each synthetic route. Therefore, all amounts of reagents used were rescaled according to the yield of each synthesis. For compounds for which no footprint data were available, such data were conveniently derived from the data of the precursors by looking at their production process. The analyses were carried out considering the entire life cycle of the different processes (from the cradle to the grave). Since limonene could be easily recovered by distillation from the reagent solution, with an experimentally verified efficiency of not less than 70%, this alternative scenario of partial limonene recovery was also considered.

### Ethical Statement

There are no ethical issues to declare.

## Conflict of Interest

The authors declare no conflict of interest.

## Author Contributions

D.P. did conceptualization, data curation, formal analysis, investigation (synthesis, optical analysis, TEM, XRD, NMR, distillation), validation, visualization (figures) and writing (original draft, review and editing). M.C. performed investigation (optical analysis), validation and writing (review and editing). M.V. did investigation (LCA) validation and writing (review and editing). L.P. did investigation (synthesis, optical analysis, XRD, NMR, distillation). E.D.G. did investigation (synthesis, optical analysis, XRD). C.L. did conceptualization, supervision, and writing (review and editing). L.M. performed conceptualization, supervision, and writing (review and editing). F.L. did conceptualization, formal analysis, investigation (synthesis, optical analysis, XRD) visualization (figures), supervision, and writing (original draft, review and editing). Contributions are assigned using CRediT taxonomy (https://credit.niso.org/).

## Supporting information



Supporting Information

## Data Availability

The data that support the findings of this study are available from the corresponding author upon reasonable request.
