# Supplementary material for: Lead Halide Perovskites Nanocrystals Synthesized in a Green, Reusable Solvent
Source: Small. 2025 May 2;21(30):2500535. doi: 10.1002/smll.202500535 (PMC12306409; doi:10.1002/smll.202500535)
Supplement: Supplementary file 1 — Supporting Information [file SMLL-21-2500535-s001.pdf]

NANO · MICRO
small

Supporting Information

for *Small*, DOI 10.1002/smll.202500535

Lead Halide Perovskites Nanocrystals Synthesized in a Green, Reusable Solvent

*Davide Pratolongo, Marta Campolucci, Marco Vocciante, Lorenzo Pugliesi, Emmanuela Di Giorgio, Chiara Lambruschini, Liberato Manna and Federico Locardi**

Supporting Information

Lead Halide Perovskites Nanocrystals Synthesized in a Green, Reusable Solvent

*Davide Pratolongo, Marta Campolucci, Marco Vocciante, Lorenzo Pugliesi, Emmanuela Di Giorgio, Chiara Lambruschini, Liberato Manna, Federico Locardi**

- **Evolution of the colour mixture during the synthesis** **S1**
- **Summary of the properties of the synthesized NCs** **S5**
- **NCs synthesized in ODE** **S6**
- **Stability of the NCs** **S8**
- **NMR results on the distilled solvent** **S10**
- **NCs obtained in a recycled solvent** **S11**
- **NMR results on the solvent stripping** **S13**
- **Balance sheet and GWP values for the dispersing/stabilizing agents** **S14**

Table S1. Evolution of the mixtures during the hot injection synthesis of $CsPbCl_3$ NCs

Solvent	T = 25 °C	T = 187 °C	Quenched reaction
**(*R*)-limonene**	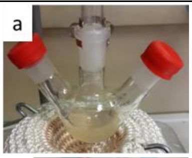	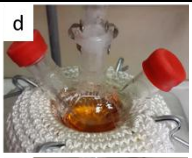	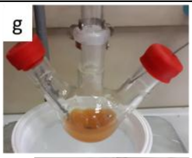
**(*S*)-limonene**	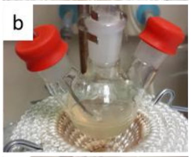	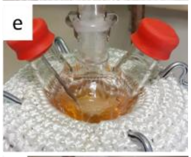	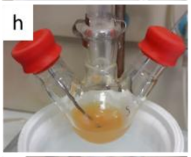
**ODE**	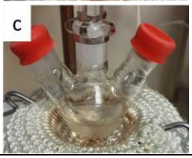	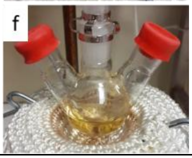	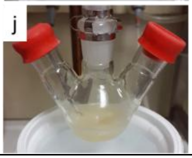

The three mixtures at room temperature appear colourless (a, b and c). Increasing the temperature the mixture in limonene turns to yellow/orange (d and e) differently from the mixture in ODE (f) which has a pale-yellow colour. After the injection, the three different mixtures become cloudy due to the precipitation of the NCs (g, h and j). The cause of the different behaviour between ODE and limonene is still under investigation; however, we hypothesize the formation of different metal complexes that confer the observed colour. These complexes probably involve the OLAM since a similar behaviour was observed in the $CsPbBr_3$ NCs synthesis (Table S2) but in the $CsPbI_3$ NCs preparation (Table S3). We also considered a possible thermal decomposition of the limonene, but this possibility was excluded according to the recovery of pure solvent after the synthesis through distillation.

Table S2. Evolution of the mixtures during the hot injection synthesis of $CsPbBr_3$ NCs

Solvent	T = 25 °C	T = 160 °C	Quenched reaction
**(*R*)-limonene**	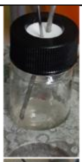	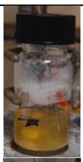	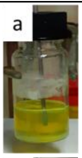
**(*S*)-limonene**	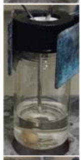	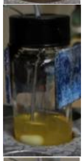	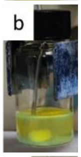
**ODE**	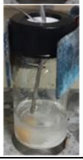	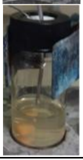	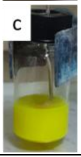

For the synthesis of the $CsPbBr_3$ NCs we observed a similar behaviour to the chloride – based NCs (Table S1). Interestingly, in limonene the final mixtures after the injection result quite transparent (a and b) whereas a cloudy suspension forms in ODE (c). The NCs can be recovered from the synthesis in ODE directly centrifuging the suspension. On the contrary, we noted that for the synthesis in limonene the addition of an antisolvent (AcOEt + 5% of ligands 10:1 OLAM:OLAC) is required to force the aggregation of the NCs and recover them by centrifugation.

Table S3. Evolution of the mixtures during the hot injection synthesis of $CsPbI_3$ NCs.

Solvent	T = 25 °C	T = 165 °C	Quenched reaction
**(*R*)-limonene**	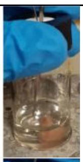	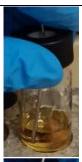	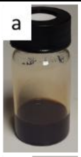
**(*S*)-limonene**	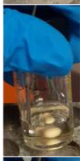	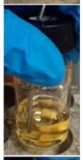	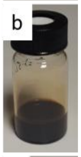
**ODE**	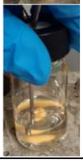	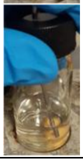	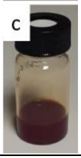

Differently for the synthesis of $CsPbCl_3$ (Table S1) and $CsPbBr_3$ NCs (Table S2) the colour and evolution of the three different mixtures were very similar. The only visible difference is in the final suspension which appeared darker for the synthesis in limonene (a and b) with respect to the ODE (c). However, this difference may be ascribed to the different NCs obtained employing the ODE (see main manuscript)

Table S4. Evaluation of the color-changing of the limonene solution with the reagents employed during the $CsPbBr_3$ NC synthesis.

Solution	T = 25 °C	T = 100 °C	T = 125 °C	T = 145 °C	T = 155 °C	T = 165 °C
**Limonene**	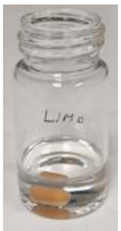	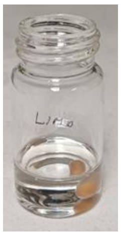	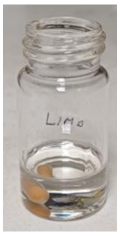	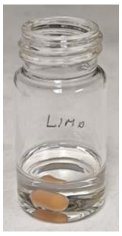	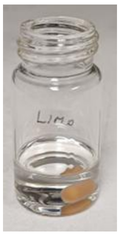	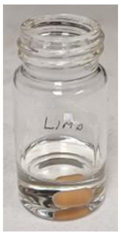
**Limonene + $PbBr_2$**	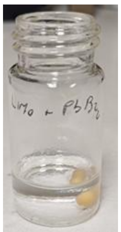	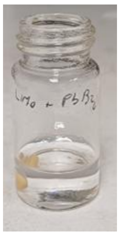	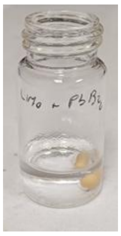	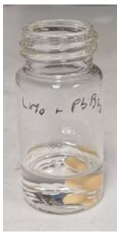	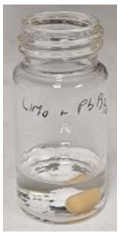	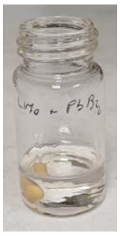
**Limonene + OLAM**	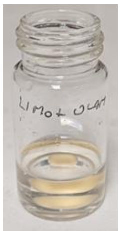	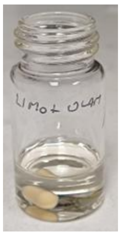	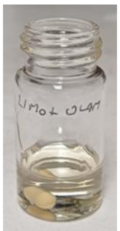	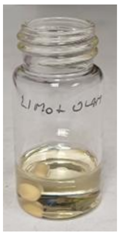	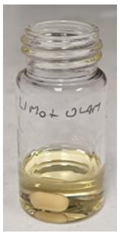	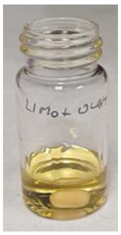
**Limonene + OLAC**	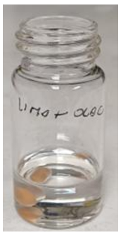	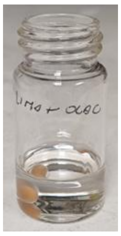	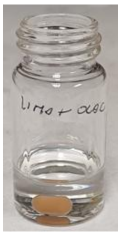	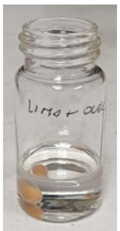	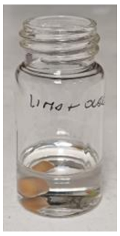	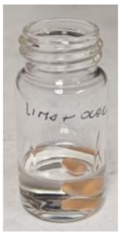
**Limonene + $PbBr_2$ + OLAM**	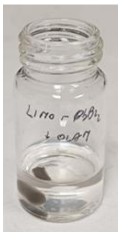	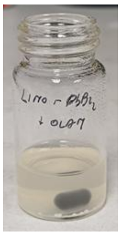	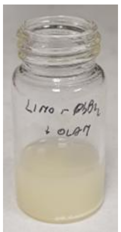	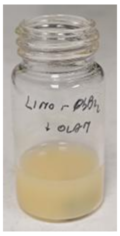	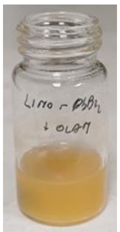	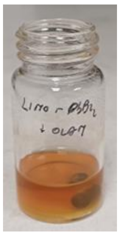
**Limonene + $PbBr_2$ + OLAC**	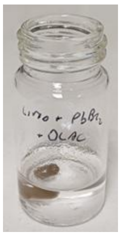	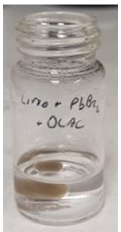	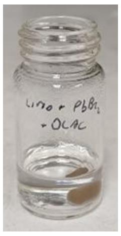	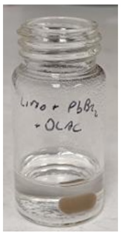	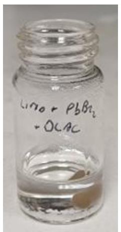	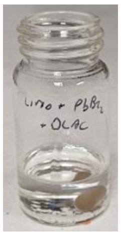

**Limonene + OLAM + OLAC**	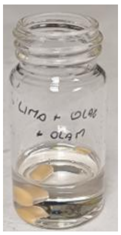	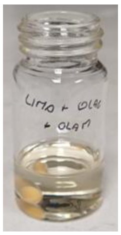	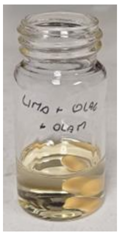	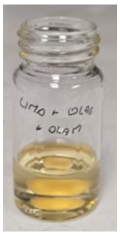	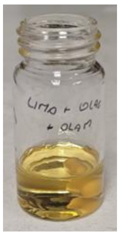	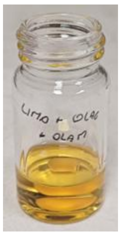

The experiments were conducted using the same quantities employed for the reported synthesis to evaluate which component of the reaction induces the color changing. From the images reported in the table, only the solutions contained OLAM (alone or mixed with $PbBr_2$ or OLAC) turn to orange. The solutions containing OLAM and OLAM + OLAC start to change the color from 125 °C to turn from pale yellow to yellow/pale orange. The $PbBr_2$ + OLAM turns the color at 100 °C from pale yellow to orange. Thus, we attributed the color changes to the presence of OLAM and the possible formation of Pb complexes.

Table S5. Comparison of the size, PLQY, emission wavelength and FWHM of the PL of the $CsPbX_3$ NCs synthetized in ODE, (*R*)- and (*S*)-limonene.

Stoichiometry	Solvent	T_{inj}	Size [nm]	PLQY	Peak wavelength	FWHM of PL
$CsPbCl_3$	ODE	200 °C	8.8 ± 1.2	34%	408 nm	79 meV
	ODE	187 °C	8.7 ± 1.0	20%	408 nm	88 meV
	(*R*)-limonene	187 °C	9.9 ± 2.5	2%	408 nm	85 meV
	(*S*)-limonene	187 °C	9.8 ± 2.5	5%	408 nm	83 meV
$CsPbBr_3$	ODE	160 °C	9.7 ± 1.0	64 %	513 nm	72 meV
	(*R*)-limonene	160 °C	9.2 ± 1.0	86%	513 nm	75 meV
	(*S*)-limonene	160 °C	9.1 ± 1.1	86%	513 nm	74 meV
$CsPbI_3$	ODE	165 °C	/	71%	690 nm	86 meV
	(*R*)-limonene	165 °C	17.0 ± 2.0	53 %	692 nm	81 meV
	(*S*)-limonene	165 °C	17.2 ± 1.8	66%	692 nm	81 meV

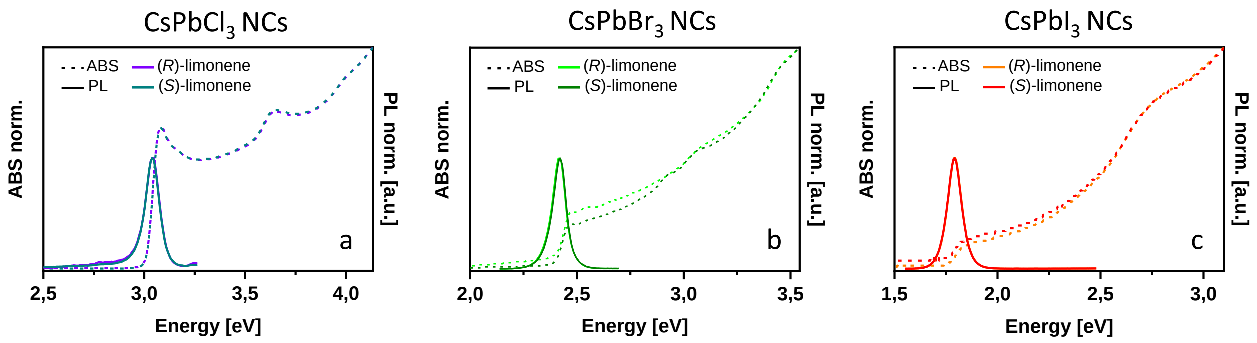

Figure S1. PL (solid line) and ABS (dash line) expressed as intensity *vs* energy of (a) $CsPbCl_3$, (b) $CsPbBr_3$ and (c) $CsPbI_3$ NCs synthetized in (*R*)- and (*S*)-limonene.

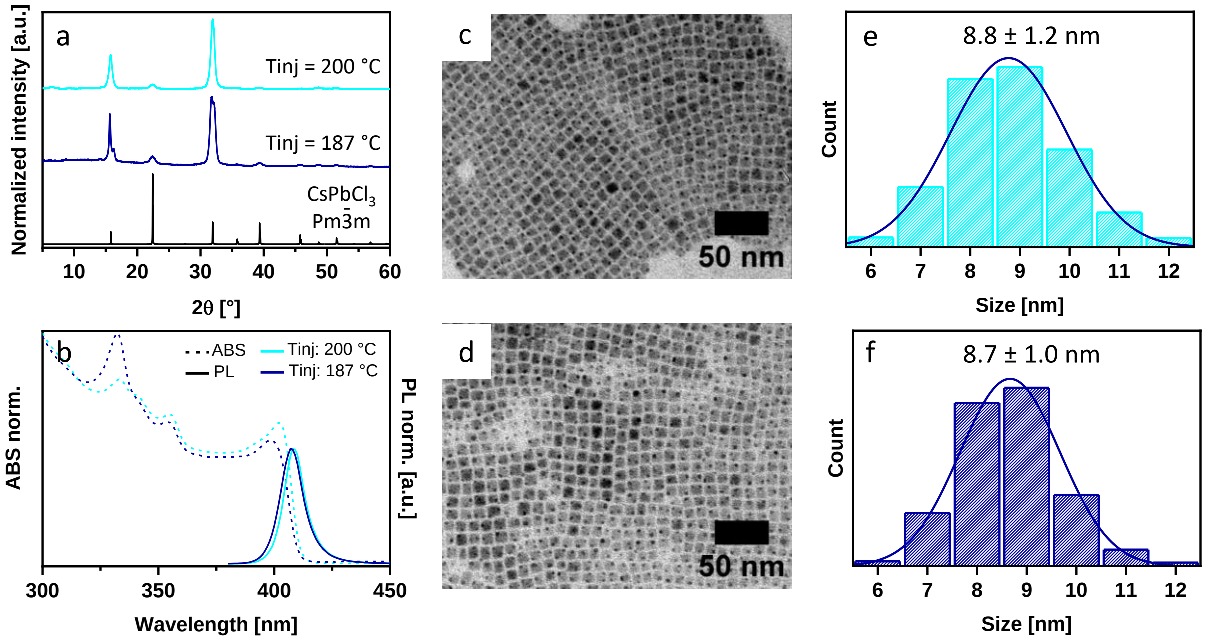

Figure S2. Comparison between the synthesis of $CsPbCl_3$ with ODE as solvent at 187 °C (line blue) and 200 °C (line cyan) in terms of (a) XRD pattern, (b) ABS and PL, (c, d) morphology and (e, f) size distribution.

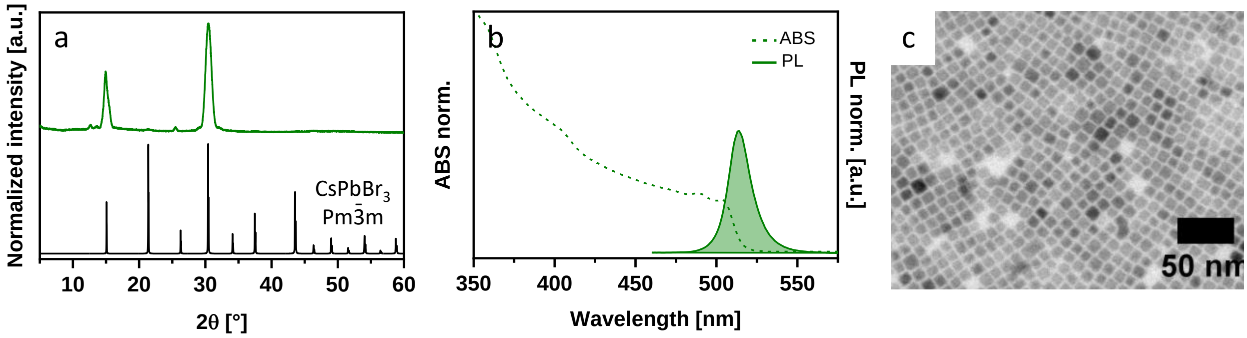

Figure S3. (a) XRD, (b) ABS (dashed line) and PL (solid line), and (c) low-resolution TEM micrography of $CsPbBr_3$ NCs synthetized in ODE with the same procedure using limonene as solvent.

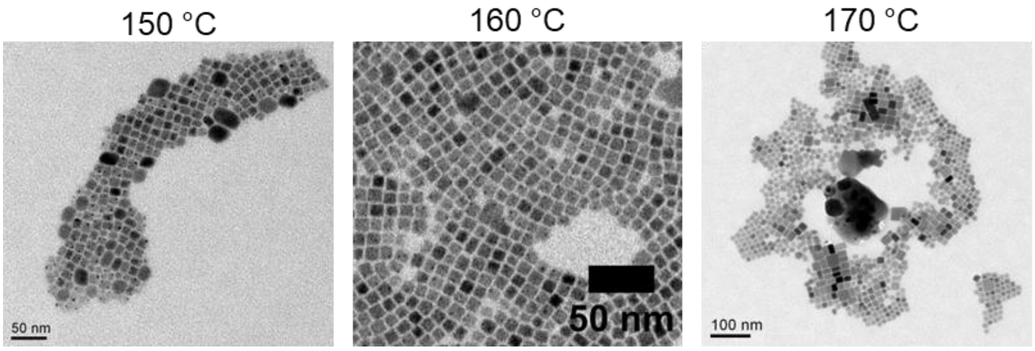

Figure S4. Low-resolution TEM micrography of $CsPbBr_3$ NCs synthetized at different temperature

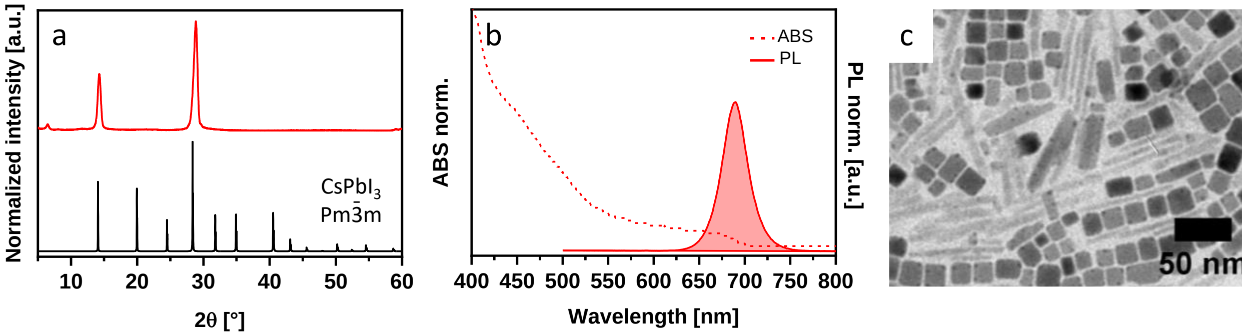

Figure S5. (a) XRD, (b) ABS (dashed line) and PL (solid line), and (c) low-resolution TEM micrography of $CsPbI_3$ NCs synthetized in ODE with the same procedure using limonene as solvent.

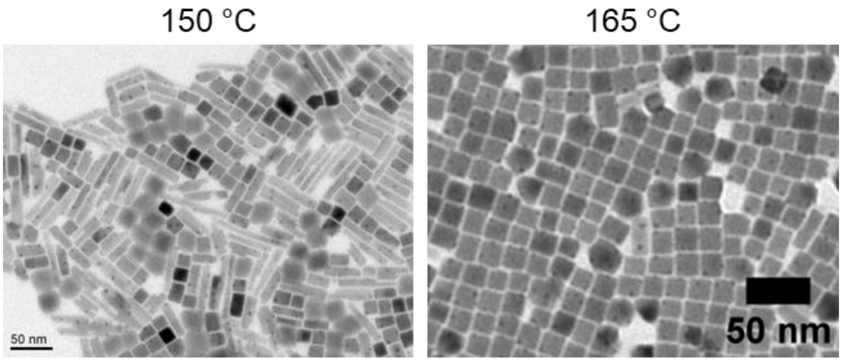

Figure S6. Low-resolution TEM micrography of $CsPbI_3$ NCs synthetized in limonene at 150 °C and 165 °C.

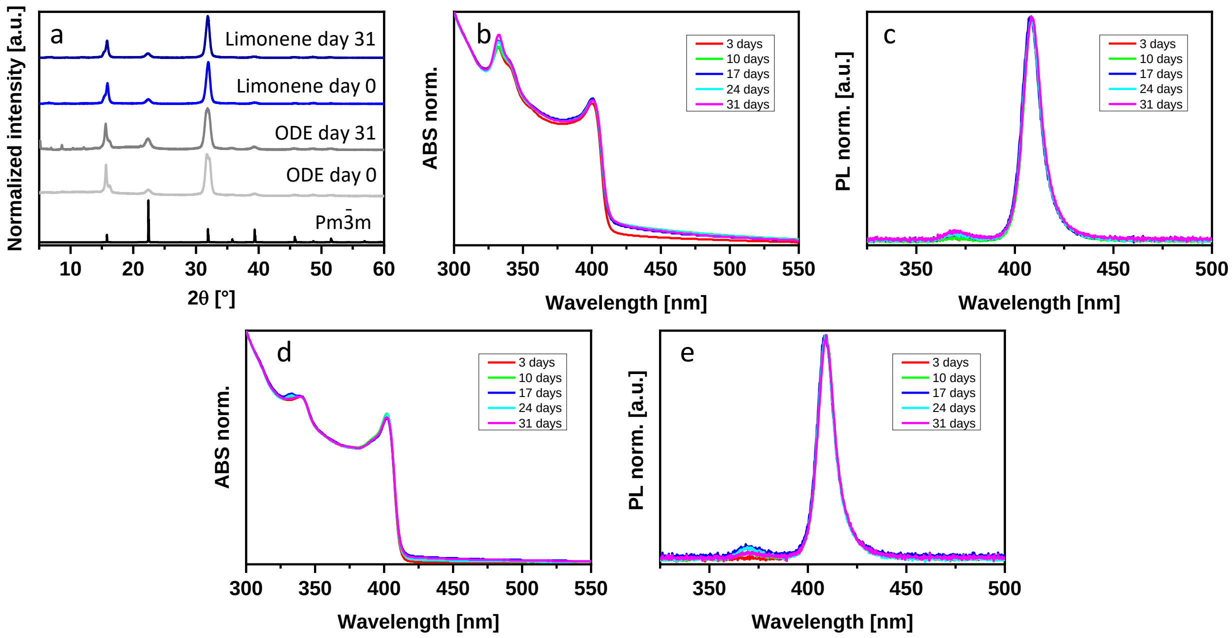

Figure S7. (a) XRD pattern of fresh $CsPbCl_3$ NCs synthetized in ODE and limonene, and after 31 days, absorbance and PL spectra of $CsPbCl_3$ synthetized in ODE (b, c) and limonene (d, e). The samples were stored under N_2 in a fridge.

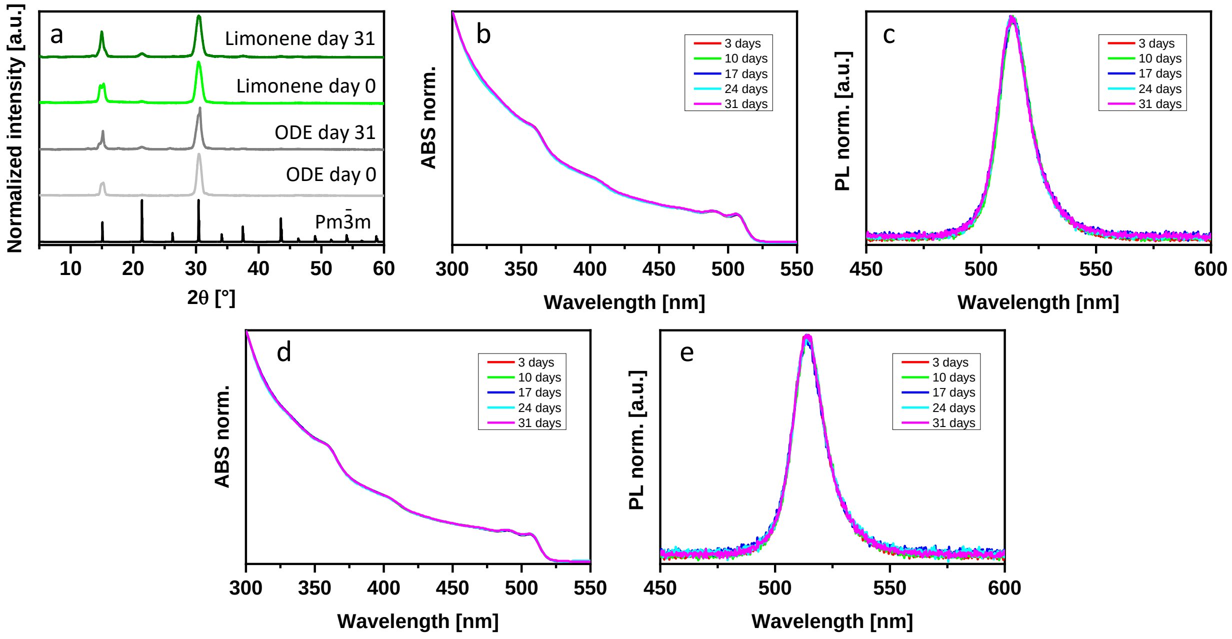

Figure S8. (a) XRD pattern of fresh $CsPbBr_3$ NCs synthetized in ODE and limonene, and after 31 days, absorbance and PL spectra of $CsPbBr_3$ synthetized in ODE (b, c) and limonene (d, e). The samples were stored under N_2 in a fridge.

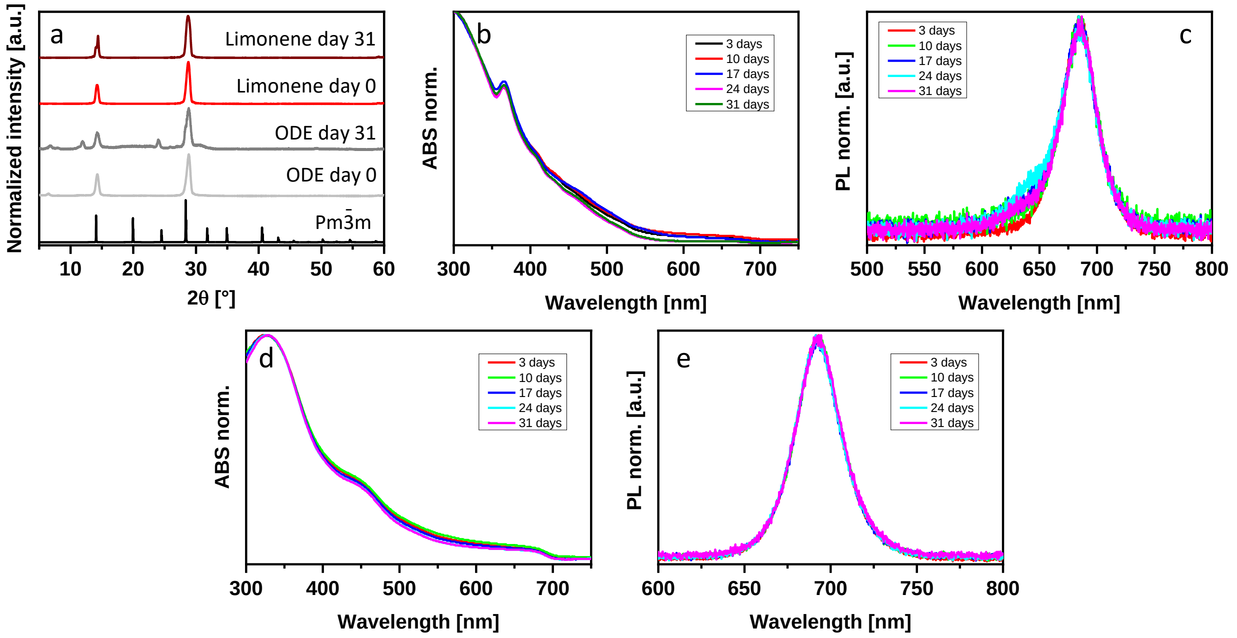

Figure S9. (a) XRD pattern of fresh $CsPbI_3$ NCs synthetized in ODE and limonene, and after 31 days, absorbance and PL spectra of $CsPbI_3$ synthetized in ODE (b, c) and limonene (d, e). The samples were stored under N_2 in a fridge.

Table S6. Comparison of the PLQY of the pristine $CsPbX_3$ NCs and after one month.

Stoichiometry	Solvent	T_{inj}	PLQY	PLQY after 31 days
$CsPbCl_3$	ODE<br>(*R*)-limonene	187 °C	34%<br>2%	27%<br>2%
$CsPbBr_3$	ODE<br>(*R*)-limonene	160 °C	64%<br>86%	53%<br>42%
$CsPbI_3$	ODE<br>(*R*)-limonene	165 °C	71%<br>53%	39%<br>38%

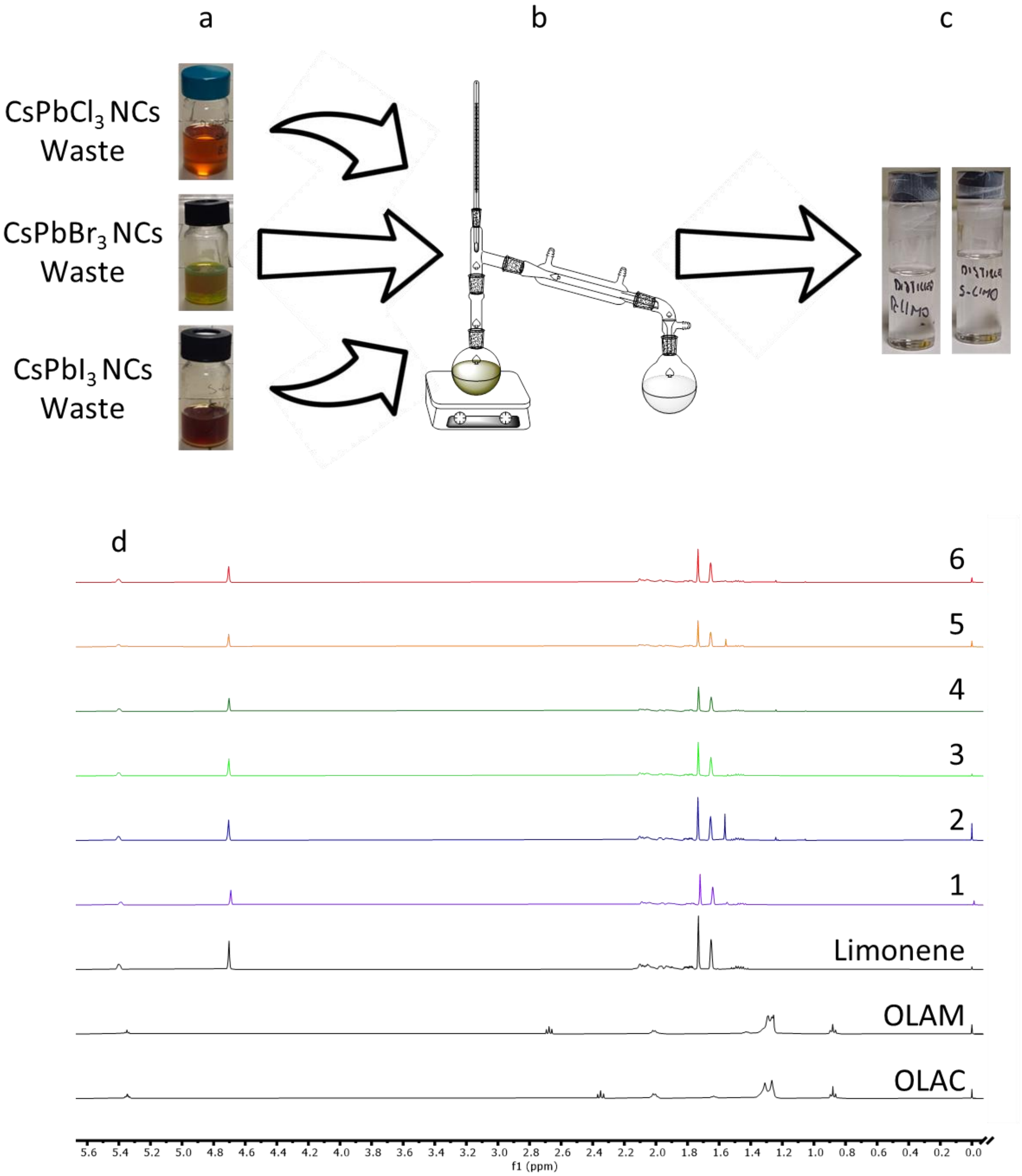

Figure S10. (a) distillation of the limonene from the synthesis waste. The waste is introduced in the distillation apparatus, distilled to obtain a colourless liquid (b) and analyzed by the acquiring ^{1}H NMR spectra using chloroform-D ($CDCl_3$) + 0.03% tetramethylsilane (TMS) V/V as deuterated solvent. Its ^{1}H NMR confirmed the structure of the limonene (c) obtained from the distillation of $CsPbCl_3$ NCs synthesis used (*R*)- (spectrum 1) and (*S*)-limonene (spectrum 2), $CsPbBr_3$ NCs synthesis with (*R*)- (spectrum 3) and (*S*)-limonene (spectrum 4) and $CsPbI_3$ NCs synthesis used (*R*)- (spectrum 5) and (*S*)-limonene (spectrum 6). Furthermore, no peaks derived from OLAM and OLAC were found, confirming the recovery of pure limonene. In spectra 2 and 5 the peak at 1.56 is ascribed to water.

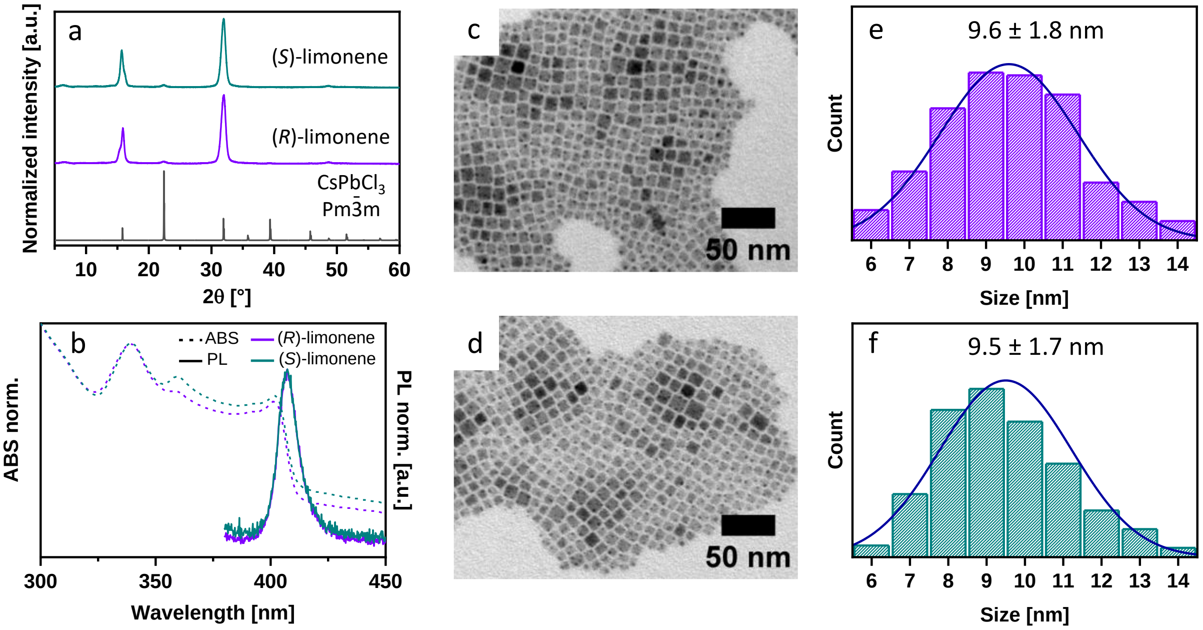

Figure S11. (a) XRD patterns, and (b) optical absorption (dash line) and PL (solid line, λexc = 365 nm) spectra of $CsPbCl_3$ NCs synthetized in distilled (*R*)-limonene (violet colour) and distilled (*S*)-limonene (blue colour); (c and d) low-resolution TEM images and (e and f) size distributions of the $CsPbCl_3$ NCs synthetized in (*R*)-limonene (c, e) and (*S*)-limonene (d, f).

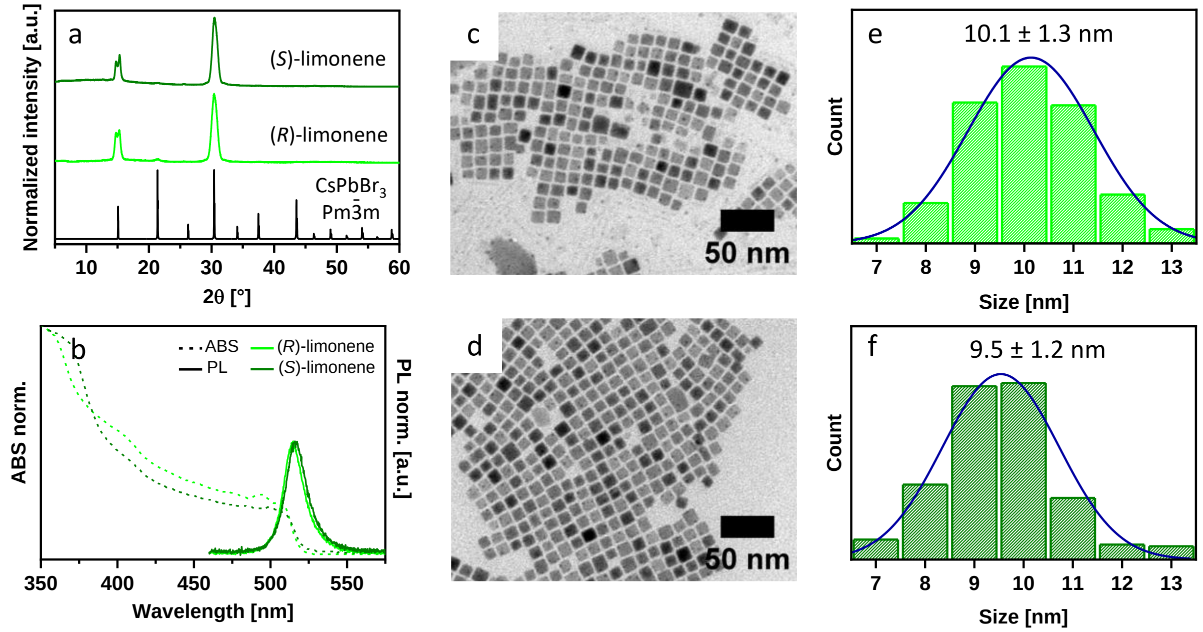

Figure S12. (a) XRD patterns, (b) optical absorption (dash line) and PL (solid line, λ_{exc} = 365 nm) spectra of $CsPbBr_3$ NCs synthetized in distilled (*R*)-limonene; (c) low-resolution TEM micrography and (d) size distribution of $CsPbBr_3$ NCs. The XRD pattern confirm the phase of the $CsPbBr_3$ NCs synthetized which have a PL peak centred at 517 nm and a mean size of 10.1 ± 1.3 nm.

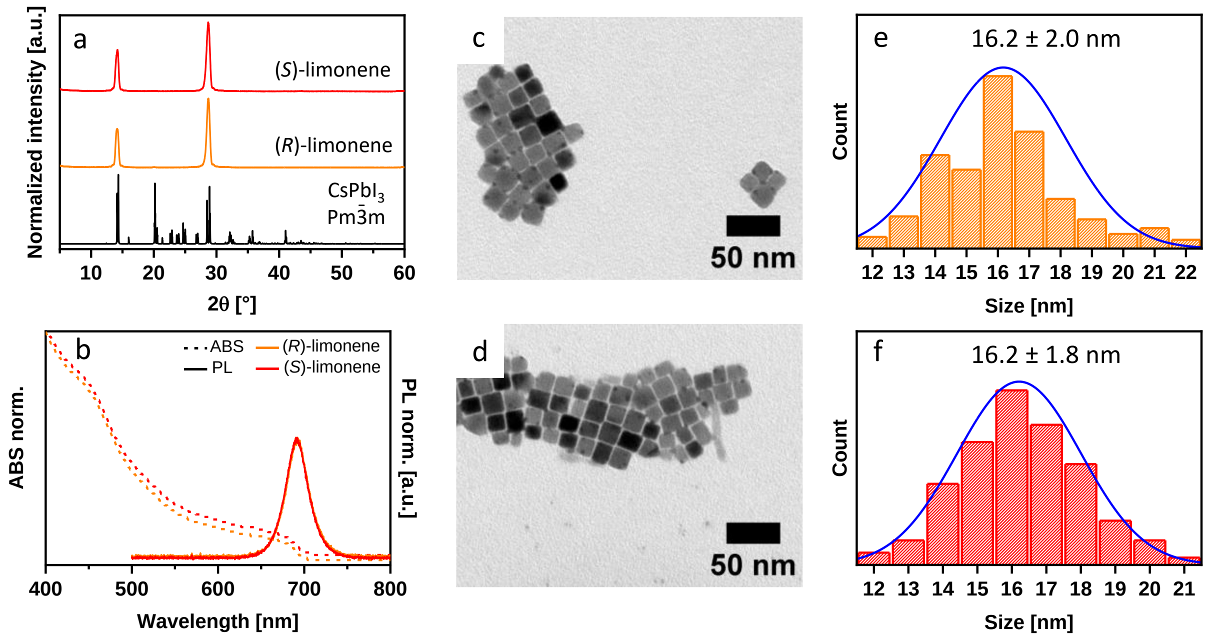

Figure S13. (a) XRD patterns, and (b) optical absorption (dash line) and PL (solid line, λexc = 365 nm) spectra of $CsPbI_3$ NCs synthetized in distilled (*R*)-limonene (orange colour) and distilled (*S*)-limonene (red colour); (c and d) low-resolution TEM images and (e and f) size distribution of the $CsPbI_3$ NCs synthetized in (*R*)-limonene (c, e) and (*S*)-limonene (d, f).

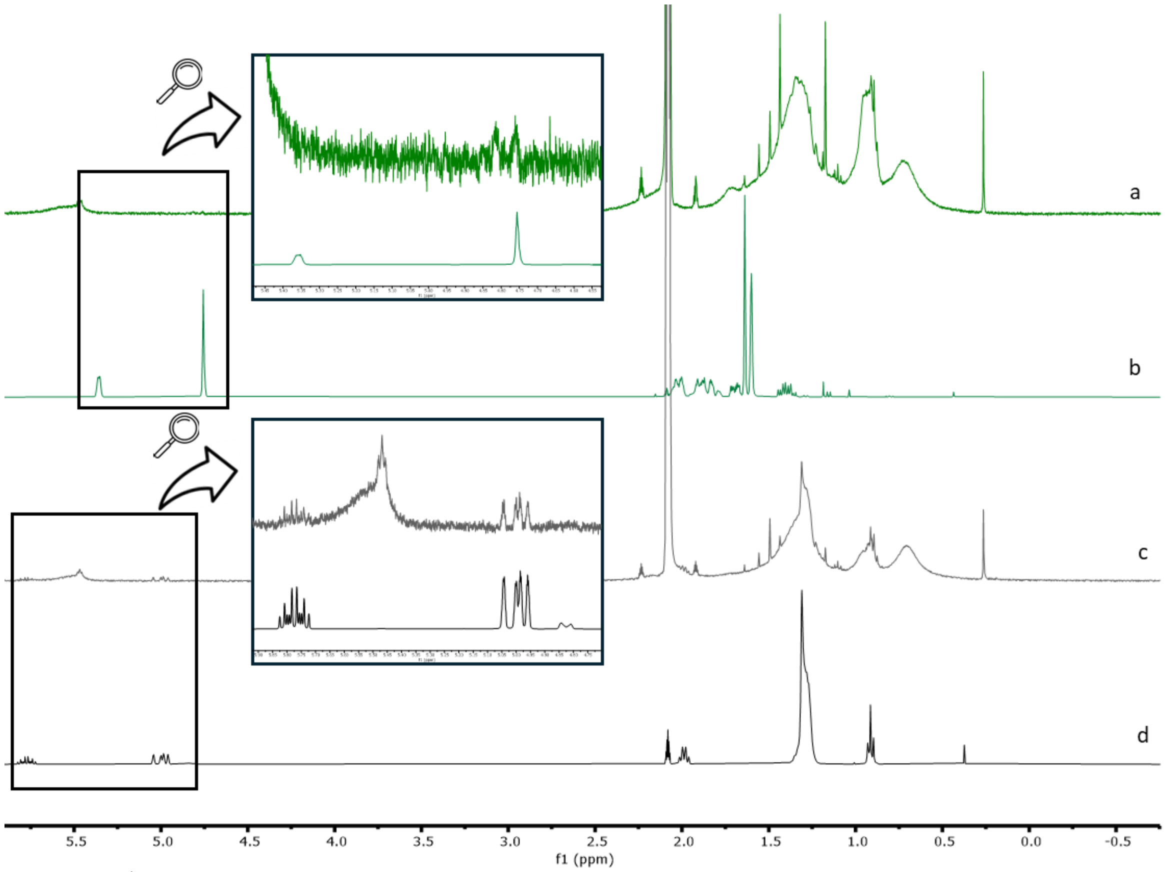

Figure S14. ^{1}H NMR of (a) $CsPbBr_3$ NCs synthesized in limonene and left under vacuum overnight, (b) pure limonene, (c) $CsPbBr_3$ NCs synthesizes in ODE and left under vacuum overnight, and (d) pure ODE. The samples were prepared by suspending the product in toluene-d8 (tol-d8). A residual of solvent is still visible for the NCs synthesized in ODE (spectrum c) whereas a complete removal of the limonene were observed (spectrum a). The sharp signal at 2.08 ppm derives from the tol-d8.

Table S7. Balance sheet of synthesis path; the normalized data refer to the production of 100g of synthetic product. In brackets are the quantities for the scenario in which limonene is recovered by distillation from the reagent solution with 70% efficiency.

			**$CsPbCl_3$**		**$CsPbBr_3$**		**$CsPbI_3$**	
**Input**			**ODE**	**LIM**	**ODE**	**LIM**	**ODE**	**LIM**
Octadec-1-ene	market for n-olefins	[L]	3.06	---	28.74	---	6.36	---
Limonene	market for citric acid	[L]	---	8.13 (2.44)	---	30.30 (9.09)	---	12.05 (3.61)
$Pb(OAc)_2*3H_2O$	derived from the market for lead, acetic acid and hydrogen peroxide as per the synthetic reaction	[g]	46.42	123.25	---	---	97.07	183.86
Cs_2CO_3	market for potassium carbonate as per affinity	[g]	9.80	26.02	---	---	20.87	39.52
Cs Oleate	derived from the market for cesium carbonate, ODE and oleic acid as per the synthetic reaction	[L]	---	---	2.87	3.03	---	---
Benzoyl chloride	market for benzyl chloride	[L]	0.13	0.34	---	---	---	---
$PbBr_2$	derived from the market for lead hy-droxide and hydrobromic acid as per the synthetic reaction	[g]	---	---	413.22	435.76	---	---
Oleyl amine iodine	market for stearic acid as per affinity	[L]	---	---	---	---	2.54	4.82
Oleyl amine (70%)	market for stearic acid as per affinity	[L]	0.18	0.49	---	---	---	---
Oleyl amine (90%)	market for stearic acid as per affinity	[L]	---	---	2.87	3.03	---	---
Oleic acid	market for stearic acid as per affinity	[L]	0.61	1.63	0.29	0.30	0.25	0.48
AcOEt	market for ethyl acetate	[L]	---	---	57.47	60.61	---	---
Toluene	market for toluene	[L]	0.55	1.46	---	---	---	---
Hexane	market for hexane	[L]	1.22	3.25	11.49	12.12	6.36	12.05
**Output**								
spent reagent solution	municipal wastewater treatment (mix)	[L]	5.76	15.30 (9.61)	103.74	109.39 (88.18)	15.52	29.40 (20.96)

Table S8. Reaction yield (expressed in mg of NCs) for the different syntheses employing ODE and limonene as solvents

	$CsPbCl_3$	$CsPbBr_3$	$CsPbI_3$
Yield in ODE	82 mg	17 mg	79 mg
Yield in Limonene	31 mg	17 mg	42 mg

Table S9. GWP values for the dispersing/stabilizing agents (see Experimental part) considering 100 g of NCs.

Material/ Operation	GWP [kg_{CO2eq} / 100g $CsPbCl_3$]		GWP [kg_{CO2eq} / 100g $CsPbBr_3$]		GWP [kg_{CO2eq} / 100g $CsPbI_3$]	
	**ODE**	**LIM**	**ODE**	**LIM**	**ODE**	**LIM**
Dispersing/ stabilizing agents	1.38	3.66	172.3	181.7	3.27	6.20
